# A cross-shear deformation for optimizing the strength and ductility of AZ31 magnesium alloys

**DOI:** 10.1038/srep29954

**Published:** 2016-07-13

**Authors:** Kotiba Hamad, Young Gun Ko

**Affiliations:** 1 School of Advanced Materials Science & Engineering, Sungkyunkwan University, Suwon 16419, South Korea; 2 Plasticity Control & Mechanical Modeling Lab., School of Materials Science & Engineering, Yeungnam University, Gyeongsan 38541, South Korea

## Abstract

Magnesium alloys have recently attracted great interest due their lightweight and high specific strength. However, because of their hexagonal close-packed structure, they have few active slip systems, resulting in poor ductility and high mechanical anisotropy at room temperature. In the present work, we used a cross-shear deformation imposed by a differential speed rolling (DSR) technique to improve the room temperature strength and ductility of AZ31 magnesium alloy sheets. To introduce the cross-shear deformation, the sheets were rotated 180° around their longitudinal axis between the adjacent passes of DSR. The sheets of the AZ31 alloy subjected to the cross-shear deformation showed a uniform fine microstructure (1.2 ± 0.1 μm) with weak basal textures. The fabricated sheets showed a simultaneous high ultimate tensile strength and elongation-to-failure, i.e., ~333 MPa and ~21%, respectively. These were explained based on the structural features evolved due to the cross-shear deformation by DSR. The high strength was attributed to the uniform fine microstructure, whereas the high ductility was explained based on the basal texture weakening.

AZ31 magnesium alloy is one alloy in the lightest class of the structural metallic materials, hence, very attractive in such applications as automotive, railway and aerospace industries. To be used for the structural applications, these materials should exhibit high strength and sufficient ductility, because the structural components are often fractured by shear or tensile forces^1^. Over recent decades, many efforts have been devoted to improve the strength of AZ31 magnesium alloys using severe plastic deformation (SPD) techniques, such as equal channel angular pressing (ECAP)^2^, accumulative roll-bonding (ARB)^3^, high pressure torsion (HPT)^4^ and asymmetric rolling (ASR)^5^. These studies indicated that the processing by SPD techniques would be preferable to obtain fine or ultrafine grained AZ31 alloys with a high strength. The improved strength of the materials after SPD deformation, however, was at the expense of decrease in the ductility, as compared to the coarse grained counterparts. For instance, an ECAP processed AZ31 alloy showed an ultimate tensile strength as high as ~370 MPa, but its total elongation was ~9%. This drawback in ductility limits the widespread structural applications of AZ31 alloys^2^. Recently, many efforts have been devoted to improve the room temperature ductility together with high strength in AZ31 alloys fabricated by SPD techniques^6^,^7^,^8^. For example, a cold pre-forging process conducted on AZ31 alloys followed by extrusion led to simultaneously increase the ultimate tensile strength and elongation-to-failure with values of 320 MPa and 19.5%, respectively^6^. The cold pre-forging process induced the formation of double-twins, which in turn led to a fine grained structure material with weak textures after the extrusion. Based on these results, the fabrication of the fine grained structure with weak basal textures was beneficial for obtaining improved mechanical properties of magnesium alloys (strength and ductility). Accordingly, several approaches were employed to introduce this kind of structural features to improve the mechanical properties of magnesium alloys. One was to impose shear bands in severely deformed materials, which should result in the texture weakening. This was recently achieved by torsional shearing deformation^9^, where the fine recrystallized grains evolved in shear bands during the torsional shearing deformation induced the weak basal texture. However, the application of these methods (SPD) to mass production can be hindered by their high cost and limited scale.

On the other hand, ASR is a promising method with potential for the continuous production of large bulk sheet materials for industrial applications. Differential speed rolling (DSR) is one form of ASR that is considered desirable for achieving fine grained structures and enhancing the mechanical properties of various materials. DSR is carried out using two rolls with identical dimensions, in which each was driven by its own motor, generating different rotation speeds between the upper and lower rolls to impose shear strain through the sheet^10^. To achieve the fine grained structure with a weak basal texture using DSR deformation at a high speed ratio of 1:4 for the lower and upper rolls, respectively, we used a cross-shear deformation, where the workpiece was rotated 180° around its longitudinal axis between the adjacent passes (hereafter called route D). Since the macro shear bands would cross each other in the subsequent number of DSR operations (Fig. 1), such a rotation technique can be beneficial for controlling the grain structure and texture of the deformed material. Our results demonstrated that the cross-shear deformation by DSR can simultaneously improve the strength and ductility of AZ31 alloys.

## Results

### Microstructural and textural features

**Figure 1:**
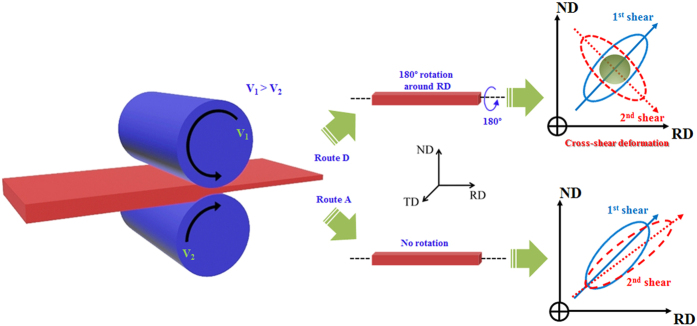
Schematic illustration showing the differential speed rolling (DSR) process and the deformations routes used in the present study (Route A and Route D “a cross-shear deformation”). Arrows inside the rolls indicate the rotational speed of the roll.

**Figure 2:**
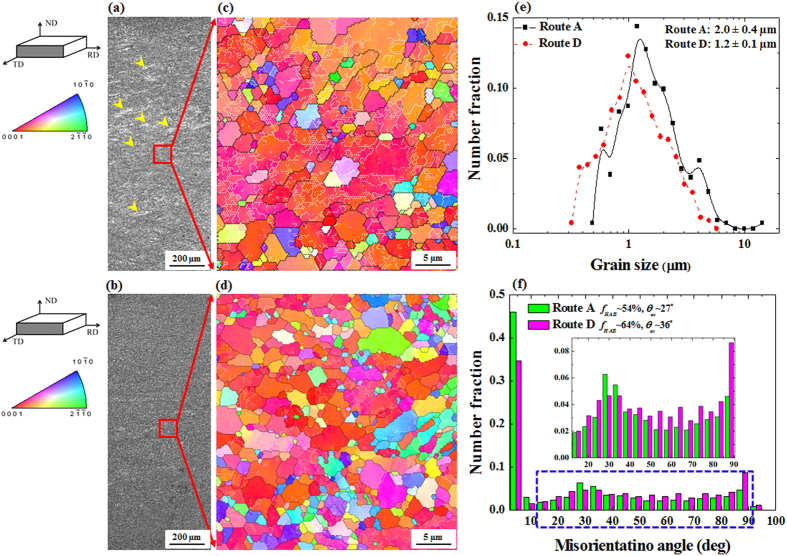
Optical micrographs showing the microstructure of the DSR-deformed AZ31 alloy samples using route A (**a**) and route D (**b**). Inverse pole figure (IPF) maps showing the microstructure of the DSR-deformed AZ31 alloy samples using route A (**c**) and route D (**d**) (white lines indicate the low angle boundaries (<15°)). Grain size distributions of the DSR-deformed AZ31 alloy samples (**e**) (Grain size distributions were obtained based on a tolerance angle of 5°). Boundaries misorientation distributions of the DSR-deformed AZ31 alloy samples showing the fraction of the high angle boundaries (*f*_*HABs*_) and the average misorientation angle (*θ*_*av*_) (**f**).

For comparison, the AZ31 alloy sample deformed by route A was also fabricated and characterized (Fig. 1). In route A, the successive rolling directions were not changed leading to a shear deformation in the same plane, as shown in Fig. 1. The effects of the cross-shear deformation (route D) on the microstructure evolution of the AZ31 alloy sample are shown in Fig. 2. Low magnification optical images taken from normal direction-rolling direction (ND-RD) planes showed that the sample fabricated by route A contained poorly refined areas, as indicated by the yellow arrows in Fig. 2a. On the other hand, a fully refined structure was achieved in the AZ31 alloy sample deformed by route D (Fig. 2b), although the total amount of deformation in both, route A and route D, were identical (total thickness reduction of 50%). In DSR deformation by route D, the sample was rotated 180° around its rolling direction between each pass. Thus, the upper side of the sample, which was in contact with the upper roll during 1^st^ pass, was altered to the lower side during 2^nd^ pass. Therefore, as the amount of strain was imparted evenly, the microstructure features were observed to be uniform across the thickness of the sample (Fig. 2b)^11^.

For more microstructural features, high magnification electron backscatter diffraction (EBSD) results taken for the samples fabricated by route A and route D are presented in Fig. 2c–f. The sample deformed by route A showed elongated grains, with an average size of 2 ± 0.4 μm, containing high fractions of low angle boundaries (LABs) (white lines in Fig. 2c,d), indicating the occurrence of deformation-induced grain subdivision. The deformation-induced grain subdivision to form fine subgrained structure would be the primary mechanism in grain refinement by means of intense straining. The inverse pole figure (IPF) map of the sample deformed using route D showed a uniform microstructure with fine grains (1.2 ± 0.1 μm) surrounded by high angle boundaries (HABs) (Fig. 2d,e). The fraction of HABs (*f*_*HABs*_) and average misorientation (*θ*_*av*_) measured from EBSD data for the DSR sample fabricated by route D exhibited higher values of ~64% and 36°, respectively, as compared to those for the sample fabricated by route A, as shown in Fig. 2f. The microstructural parameters of the sample subjected to the cross-shear deformation (route D) strongly suggested the evolution of dynamic recrystallized (DRX) grains, where during the dynamic recrystallization; there will be progressive increase in boundary misorientations and conversion of LABs into HABs^12^. The cross-shear deformation can induce the formation of preferred nucleation sites for dynamic recrystallization during the DSR processing, leading to an increase in the fraction of DRX grains. It has been established that the driving force for nucleation and growth during hot deformation was related to the reduction in strain energy and strain gradient. Due to the large strain gradient in shear bands formed in grain interior, these bands would act as nucleation sites^8^. Accordingly, the cross-shear deformation (route D) that caused the intersection of shear bands would create more sites for nucleation and lead to a high fraction of DRX grains during DSR deformation. On the other hand, the shear deformation introduced into the same plane (route A) led mainly to the accumulation of LABs rather than the evolution of DRX grains.

**Figure 3:**
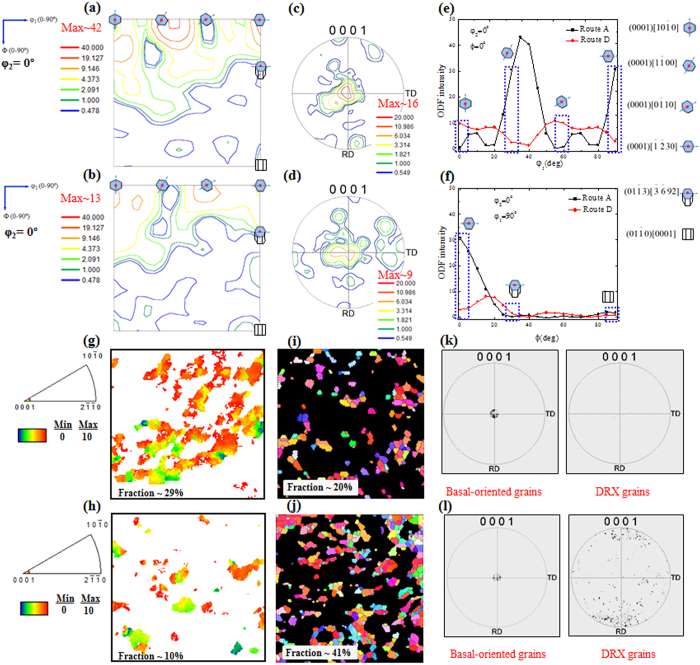
Orientation distribution functions (ODF) and (0001) pole figures (PF) of the DSR-deformed AZ31 alloy samples using route A (**a,c**) and route D (**b,d**). ODF intensity distributions of the DSR-deformed AZ31 alloy samples showing the basal texture components along φ_1_ (φ_2_ = 0°, Φ = 0°) (**e**) and along Φ (φ_2_ = 0°, φ_1_ = 90°) (**f**). Partitioned maps showing basal-oriented grains and dynamic recrystallized (DRX) grains, and the (0001) pole figures related to these maps for the DSR-deformed AZ31 alloy samples using route A (**g,i,k**) and route D (**h,j,l**).

Orientation distribution functions (ODF) and pole figures (PF) determined by EBSD indicated that the deformed samples (route A and route D samples) exhibited basal textures with most of the (0001) plane oriented toward the ND (Fig. 3a–d). To clarify the evolution of the basal texture components, the ODF intensity distributions of the deformed samples along φ_1_ and Φ in the reduced Euler space (φ_2_: 0°, φ_1_:0–90°, Φ:0–90°) are shown in Fig. 3e,f. The weaker basal texture components including (0001)[10
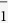
0], (0001)[1
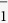
00], (0001)[0
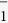
10] and (0001)[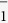

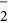
30], in the sample deformed by route D as compared to those in the sample deformed by route A were related to the high fraction of DRX grains evolved due to the cross-shear deformation induced by route D^8^,^13^,^14^. It is well-known that DRX grains are oriented randomly at the expense of (0001)//ND-oriented grains (basal-oriented grains), which in turn leads to a basal texture weakening. The partitioned maps and related textural characteristics of the basal-oriented grains and DRX grains in the DSR-deformed AZ31 alloy samples are shown in Fig. 3 (3g-k for route A and 3h-l for route D). It could be noted that the fraction of the basal-oriented grains in the sample deformed by route A (~29%) was higher than that in the sample deformed by route D (~10%). This was conjugated with a low fraction of the DRX grains (~20% in the sample deformed by route A) as compared to that of the sample deformed by route D (~41%). In addition, the intensity of the random texture related to the DRX grains evolved in the sample deformed by route D was pronounced, as shown by the (0001) pole figure obtained from the map of the DRX grains (Fig. 3j,l).

### The combination of high strength and high ductility

**Figure 4:**
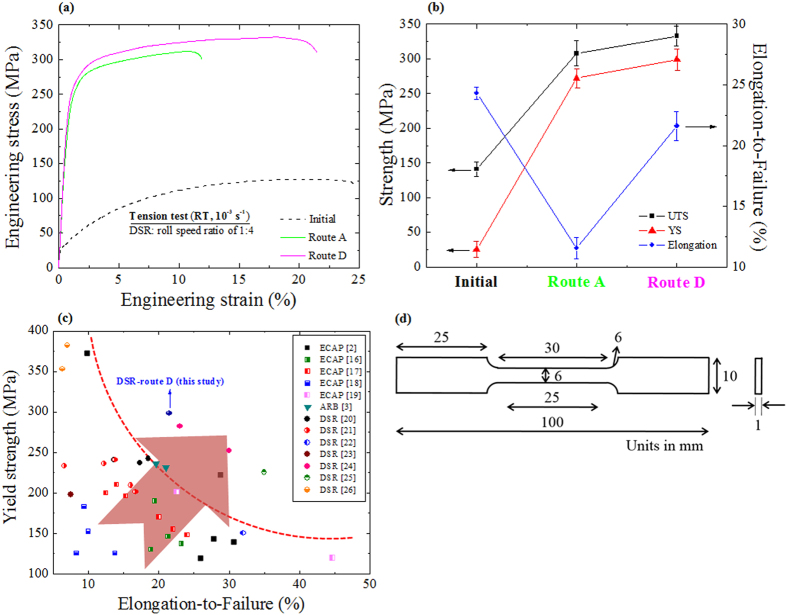
Room temperature engineering stress-engineering strain curves of the initial AZ31 alloy sample (not deformed) and the DSR-deformed AZ31 alloy samples (route A and route D) (**a**). Yield strength (YS), ultimate tensile strength (UTS) and elongation-to-failure of the initial AZ31 alloy sample (not deformed) and the DSR-deformed AZ31 alloy samples using route A and route D (**b**). Yield strength versus elongation-to-failure of AZ31 alloys deformed by equal channel angular pressing (ECAP)^2^,^16^,^17^,^18^,^19^, accumulative roll-bonding (ARB)^3^ and differential speed rolling (DSR)^20^,^21^,^22^,^23^,^24^,^25^,^26^ (**c**). Specimens geometry used for the tensile tests (**d**).

Figure 4a presents the data obtained from tension tests conducted at room temperature and a strain rate of 10^−3^ s^−1^ of the initial AZ31 alloy sample (not deformed) and the samples fabricated by route A and route D. A summary of yield strength (YS), ultimate tensile strength (UTS) and elongation-to-failure values for these samples are also shown in Fig. 4b. The YS, UTS and elongation-to-failure of the initial sample were measured to be ~35, ~125 MPa and ~25%, respectively. The high values of YS and UTS obtained for the DSR-deformed AZ31 alloy samples were in good agreement with the microstructures evolved after the deformation (Fig. 2). In addition, the elongation-to-failure value of the sample processed by route A (~11%) was lower than that of the initial sample. In general, the fine grained metallic materials fabricated via SPD suffer from very limited ductility^2^, whereas materials with a coarse grained structure exhibit poor strength conjugated with high ductility^15^. On the other hand, the sample deformed by route D showed a high value of elongation-to-failure (~21%), which was close to that of the initial sample (~25%), although the grain size of this sample (route D) was finer than those of the initial sample and the sample deformed by route A. Therefore, it is worth noting that the AZ31 alloy sample subjected to the cross-shear deformation (route D) exhibits a combination of high strength and improved ductility.

### Comparison with other SPD techniques

To compare our results fairly with the mechanical properties of AZ31 alloys deformed by other SPD techniques, we have collected tensile data, available from literatures, for AZ31 alloy materials fabricated by ECAP^2^,^16^,^17^,^18^,^19^, ARB^3^ and DSR^20^,^21^,^22^,^23^,^24^,^25^,^26^, as shown in Fig. 4c. It is clearly seen from Fig. 4c that most of the literature data were located between the left-upper and right-bottom corners, showing an inverse relationship between the strength and ductility. However, some other data, including the result obtained for the sample subjected to the cross-shear deformation, stood out from that trend, suggesting the superior combination of the mechanical properties (strength and ductility).

## Discussion

In recent years, AZ31 magnesium alloys have received a significant attention in the automotive and electronics industries due to a number of advantages that they offer, such as high specific strength and specific modulus. Although the microstructure of AZ31 alloys can be greatly refined using severe plastic methods, the limited ductility of fine grained AZ31 alloys has emerged as a particularly challenging issue in the study and application of this class of materials^26^. In this study, we used a processing route for AZ31 alloys (Fig. 1) which resulted in the superior combination of the mechanical properties (strength and ductility) (Fig. 4), in contrast to the typical inverse relationship between the strength and ductility.

The mechanical properties of bulk materials are mainly controlled by their microstructure and texture. As shown from the EBSD results presented above, the microstructure of the AZ31 alloy sample deformed by DSR using route D composed of fine grains (1.2 ± 0.1 μm) surrounded by HABs (>15°). In addition, the high fraction of randomly-oriented fine grains (DRX grains) obtained by the cross-shear deformation, led to a weak basal texture in the sample deformed by route D. Due to the cross-shear deformation between the DSR passes, preferential nucleation sites for dynamic recrystallization are formed, leading to significant changes in the grain structure and texture of the processed AZ31 alloy. Accordingly, the grain refinement and texture changes would be discussed to explain the simultaneous improvement of strength and ductility of the DSR-deformed AZ31 alloy samples.

**Figure 5:**
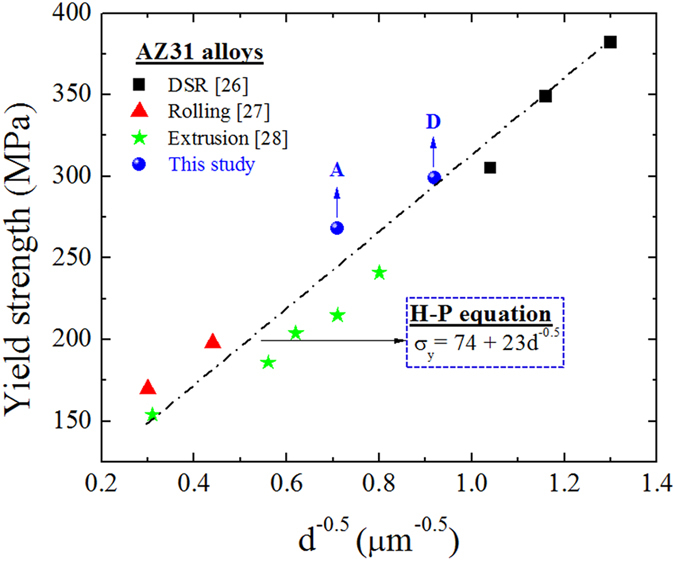
The Hall-Petch relationship for AZ31 alloys deformed by differential speed rolling (DSR)^26^, rolling^27^ and extrusion^28^.

The significant improvement in the strength after the second rolling pass by route D was mainly due to the grain refinement, induced by the transformation from coarse grains to the fine grains, i.e. the Hall-Petch (H-P) effect (Fig. 5). Based on H-P equation (σ_*y*_ = σ_0_ + *K*_*y*_*d*^−0.5^), the fine grains have limited capacity for dislocation accumulation, where *σ*_*y*_ is the yield stress, *σ*_*0*_ is the friction stress when dislocations move on the slip plane, d is the grain size and *K*_*y*_ is a factor related to the stress concentration. Here, we consider that the grain refinement was the dominate factor responsible for the high strength of the AZ31 alloys deformed by DSR, where the increment of the yield stress by grain-size strengthening can be estimated by the H-P equation presented above. To confirm the grain-size strengthening, the H-P equation constructed for the AZ31 alloy samples processed by DSR^26^ and by other deformation paths, such as rolling^27^ and extrusion^28^, was presented in Fig. 5. It is clearly seen from Fig. 5 that all of the data fitted well into a single straight line based on the H-P equation. According the straight line, *σ*_*0*_ and *K*_*y*_ were found to be 74 MPa and 23 MPa (m)^0.5^, respectively.

**Figure 6:**
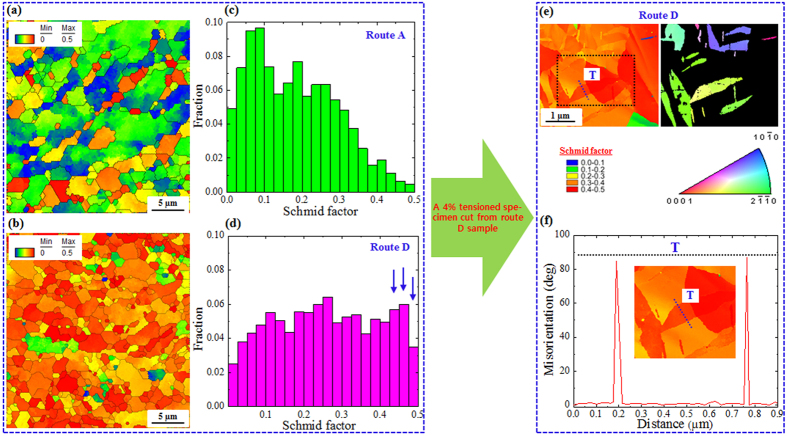
Schmid factor maps and distributions of the DSR-deformed AZ31 alloy samples using route A (**a,c**) and route D (**b,d**). Schmid factor and partitioned {10
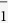
2} twins (tension twins) maps obtained from EBSD data of a 4% pre-strained tensile specimen cut from the DSR-deformed AZ31 alloy sample using route D (**e**). Misorientation profile showing point-to-point misorientations along the {10
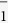
2} twin (**f**).

In general, the ductility decreases with decreasing grain size under room temperature tensile tests. However, the ductility of the AZ31 alloy sample subjected to the cross-shear deformation (route D) increased with decreasing grain size (Figs 2 and 4). This tendency was mainly related to the texture evolution in the AZ31 alloy sample after the DSR deformation by route D. As shown by Fig. 3, the textures evolved after the DSR deformation (route A and route D) could be simply represented by basal components, which are well-known texture components that form during various rolling procedures of hexagonal close-packed materials. The rolling procedure used in this study (cross-shear deformation), accordingly, resulted in basal texture components, but with a low maximum intensity, as shown in Fig. 3b–f. This was mainly attributed to the evolution of randomly-oriented grains (DRX grains) due to the cross-shear deformation. The weak components evolved due to the dynamic recrystallization during the deformation by route D led to a texture softening. This was confirmed by the Schmid factor calculations of the (0001)[11
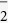
0] slip system along RD as a tensile axis (Fig. 6). Based on the calculated Schmid factor distributions for the (0001)[11
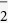
0] slip system (Fig. 6), the distribution obtained for the sample with the weak basal texture (route D) tended to shift toward high values of the Schmid factor, as indicated by the arrows in Fig. 6d. It is well-known that extension twins ({10
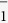
2} twins), which are primarily activated within the first stages of tensile deformation, can be easily formed in the grains with a high Schmid factor (>0.3)^29^. Hence, these grains can accommodate larger amounts of deformation as compared to those with low factors. The evolution of tension twins in the grains with the Schmid factor higher than 0.3 was shown by EBSD results of a 4% pre-strained tensile specimen cut from the AZ31 alloy sample deformed by route D (Fig. 6e,f). Extension twins, which usually lead to an 86° reorientation of grains (Fig. 6f)^30^, were observed in almost all of the grains and they were successfully induced by the pre-straining (4%), as shown by Fig. 6e.

Although the texture softening resulted from the increase in the Schmid factors on the basal plane can reduce the strength, the date obtained for the present sample fitted well with the H-P equation (Fig. 5), indicating that the strength would be increased by taking the full advantage of grain size refinement. Accordingly, the strength enhancement with the corresponding increase in ductility of the AZ31 alloy sample subjected to the cross-shear deformation was obtained through a combination of grain refinement and texture control.

**Figure 7:**
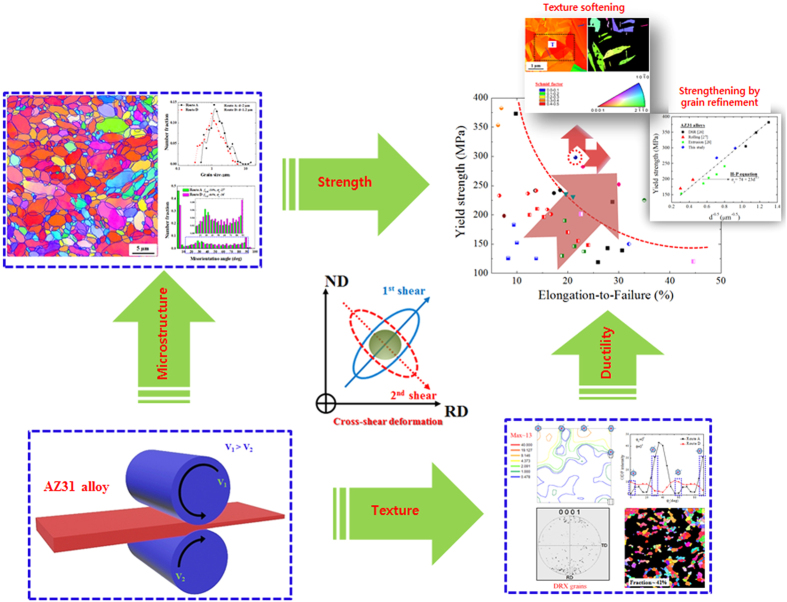
Microstructure, texture and room temperature tensile properties of the AZ31 alloy sample deformed by route D.

To summarize, the effects of the cross-shear deformation on microstructure, texture and room temperature tensile properties of the AZ31 alloy sample were presented in Fig. 7. We have applied a cross-shear deformation to AZ31 alloy sheets by DSR to achieve simultaneously enhanced tensile properties at room temperature. We have shown that the cross-shear deformation can produce uniform sheet materials with a high ultimate strength and ideal ductility of ~330 MPa and ~21%, respectively. The high strength was attributed largely to the formation of uniform fine microstructure with an average grain size of ~1.2 ± 0.1 μm. In the addition, the high ductility of the AZ31 alloy sheets with the fine grained microstructure was due to the weak basal texture evolved after the DSR deformation. This texture can enhance the formation of {10
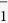
2} twins during the earlier stages of the tensile deformation, which in turn serves as a complementary deformation mechanism to enhance the ductility. These results strongly suggest that the processing by DSR is a potential technique for controlling the microstructure and texture of difficult-to-deform metals and correspondingly for improving their performance.

## Methods

### Materials fabrication

The material used in this study was AZ31 magnesium alloy sheets with a chemical composition (in wt. %) of 2.89 Al, 0.96 Zn, 0.31 Mn, 0.15 Fe, 0.12 Si, and balance Mg. The as received samples were homogenized for 24 h at 673 K and cooled in air to obtain a fully annealed microstructure with equiaxed grains (not shown here). Before DSR deformation, the sheets were machined into plate-type samples with dimensions of 70 × 30 × 4 mm. A series of DSR operations were performed at 423 K using two working rolls with an identical diameter of 220 mm, which revolved at a roll speed ratio of 1:4 for the lower and upper rolls, respectively, under the condition that the velocity of the lower roll was fixed to ~5 m/min. Two DSR passes were carried out with a thickness reduction per pass of 30%, so that a total thickness reduction was ~50% after 2^nd^ pass of DSR. To introduce a cross-shear deformation, the samples were rotated 180° around their longitudinal axis between the adjacent passes, as shown in Fig. 1.

### Structural features and mechanical properties

The microstructural and textural features of the AZ31 alloy samples were examined by electron backscatter diffraction (EBSD) in a scanning electron microscope with a field-emission gun (Hitachi S-4300 FESEM). The data was analyzed using TSL OIM 6.1.3 software. Sheets cut from the RD-ND plane of the fabricated samples for EBSD analysis were polished mechanically and etched in a 2% solution of nitric acid in ethanol. EBSD scans at high magnifications were obtained using step size of 0.02 μm. This step size was suitable for accurate grain boundaries analysis. The grain boundary distribution was evaluated under the assumption of a 2 to 15° misorientation angle for low angle boundaries (LABs) and above 15° for high angle boundaries (HABs). For texture evolution, the data from the EBSD experiments was analyzed by the orientation distribution functions (ODF) calculated using a Harmonic Series Expansion method. The analysis was carried out in an Euler angle space (φ_1_ = 0–90°, Φ = 0–90°, and φ_2_ = 0°) using the non-orthonormal sample symmetry. The fraction of HABs, boundary misorientation distributions, grains size distributions and texture obtained from the EBSD data were averaged over at least three maps including ~400 grains. For tension tests, the dog-bone specimens with a gauge length of 25 mm were cut along the RD (Fig. 4d), and an initial strain rate was determined to be 10^−3^ s^−1^.

## Additional Information

**How to cite this article**: Hamad, K. and Ko, Y. G. A cross-shear deformation for optimizing the strength and ductility of AZ31 magnesium alloys. *Sci. Rep.* **6**, 29954; doi: 10.1038/srep29954 (2016).
